# Comparative Anatomy of the Hind Limb Vessels of the Bearded Capuchins (*Sapajus libidinosus*) with Apes, Baboons, and *Cebus capucinus*: With Comments on the Vessels' Role in Bipedalism

**DOI:** 10.1155/2013/737358

**Published:** 2013-12-12

**Authors:** Roqueline A. G. M. F. Aversi-Ferreira, Tainá de Abreu, Gabriel A. Pfrimer, Sylla F. Silva, Janine M. Ziermann, Frederico O. Carneiro-e-Silva, Carlos Tomaz, Maria Clotilde H. Tavares, Rafael S. Maior, Tales A. Aversi-Ferreira

**Affiliations:** ^1^Department of Anatomy, Howard University College of Medicine, 520 W Street NW, Numa Adams Building, Washington, DC 20059, USA; ^2^Laboratory of Anthropology, Biochemistry, Neuroscience and Primates' Behavior (LABINECOP), Federal University of Tocantins, Avenida NS 15 ALC NO 14 109 Norte, 77001-090 Palmas, TO, Brazil; ^3^Graduate School of Animal Biology, Institute of Biology, University of Brasilia, Darcy Ribeiro Campus, 70910-900 Brasília, DF, Brazil; ^4^Department of Physiology, Laboratory of Neuroscience and Behavior, University of Brasilia, Darcy Ribeiro Campus, 70910-900 Brasília, DF, Brazil; ^5^Graduate School of Veterinary, Federal University of Uberlândia, Rua Ceará S/N Bloco 2D Campus Umuarama, 38400-902 Uberlândia, MG, Brazil

## Abstract

Capuchin monkeys are known to exhibit sporadic bipedalism while performing specific tasks, such as cracking nuts. The bipedal posture and locomotion cause an increase in the metabolic cost and therefore increased blood supply to lower limbs is necessary. Here, we present a detailed anatomical description of the capuchin arteries and veins of the pelvic limb of *Sapajus libidinosus* in comparison with other primates. The arterial pattern of the bearded capuchin hind limb is more similar to other quadrupedal *Cebus* species. Similarities were also found to the pattern observed in the quadruped *Papio*, which is probably due to a comparable pelvis and the presence of the tail. *Sapajus*' traits show fewer similarities when compared to great apes and modern humans. Moreover, the bearded capuchin showed unique patterns for the femoral and the short saphenous veins. Although this species switches easily from quadrupedal to bipedal postures, our results indicate that the bearded capuchin has no specific or differential features that support extended bipedal posture and locomotion. Thus, the explanation for the behavioral differences found among capuchin genera probably includes other aspects of their physiology.

## 1. Introduction

There are numerous studies about *Sapajus* behavior, cognition, and bipedalism [1–5], but the anatomical descriptions of these species are scarce in scientific literature and the majority of those works concentrate on thoracic limbs [6–13]. Few descriptions are related to the nervous system of the hind limbs [14, 15]. It is not surprising that the anatomical descriptions available of the lower limbs that support the data about its bipedalism focusing mainly on muscles and bones, although these structures are supplied by the arterial system.

Indeed, some studies have focused on the kinematics of capuchin bipedalism [16] but few have addressed the metabolic cost of bipedal gait on hind limbs. Although this behavior requires higher energetic cost on hind limbs [17] and depends on an adequate arterial supply to muscles, only a handful of articles have emphasized the anatomical features of capuchin hind limbs vessels so far. Brown [18], for example, described the femoral artery of the apes and also mentioned *Cebus* with regard to the saphenous arteries. Manners-Smith [19] investigated the hind limb arteries of primates and compared two specimens of *Cebus capucinus* to other primates of different families, that is, Simiidae, Cercopithecidae, Hapalidae, and Prosimiae. Among New World primates, however, this work focused mostly on the genus *Ateles*. There were only few superficial descriptions of *Cebus capucinus'* vessels and no pictures were provided for this species. Furthermore, neither of the authors mentioned above [18, 19] described the arteries supplying the muscles.

Indeed, the anatomical aspects of hind limb vessels could have considerable importance to keep the muscles working to support the weight when the animal is on a bipedal gait, because the more ramified arterial system could increase the area for the distribution of blood [20, 21]. Applying this rationale to different primate species, the comparative anatomical description of vessel plays important role to provide data about the physiology and behavior behind bipedalism.

Therefore, the main objective of this study was to generate modern and accurate data about the anatomical pattern of the vessels of *Sapajus libidinosus* (bearded capuchin) hind limbs and compare our findings with the anatomy of others species available in the scientific literature, namely, *Cebus capucinus* [19, 22], apes [23], baboons [24, 25], and modern humans [26].

We analyzed the vessels of the hind limbs of bearded capuchins, considering aspects as origin, distribution, and irrigation. We are discussing our results in comparison with other species and present our formatted data in a table. Moreover, we made an effort to identify associations of our data that would support the physiological anatomy of the bipedal position.

## 2. Material and Methods

### 2.1. Samples

Nine adult bearded capuchin specimens (three females, six males) were used in this study. Their weights were between one and four kilograms. No animal was killed for the purposes of this study; five of them suffered accidental deaths in their natural habitats and were deposited in the anatomical collection of the Anthropology, Biochemistry, Neuroscience and Behavior of Primates Laboratory (LABINECOP) from the Federal University of Tocantins, Tocantins State, Brazil. The other four belonged to the Brazilian Institute of Environment and Renewable Natural Resources (IBAMA) archive and were donated for studies in the 1970s. This work was approved by the Institutional Ethical Committee from the Federal University of Goiás, Goiás State, Brazil (CoEP-UFG 81/2008, authorization from the IBAMA number 15275).

### 2.2. Preparation of the Animals for Dissection

All procedures involving the animals were done in accordance with the guidelines of the Brazilian Society of Animal Experimentation (COBEA). After the trichotomy with a razor blade, the animals received perfusion, by the femoral artery, with 10% of formaldehyde for fixation. The animals were conserved in 10% of formaldehyde, in covered opaque cubes.

### 2.3. Nomenclature

We used the human nomenclature for the vessels whenever possible. In the cases where no human nomenclature was available we followed the terminology of [23].

## 3. Results

### 3.1. Arterial Description (Table 1)

**Figure 1:**
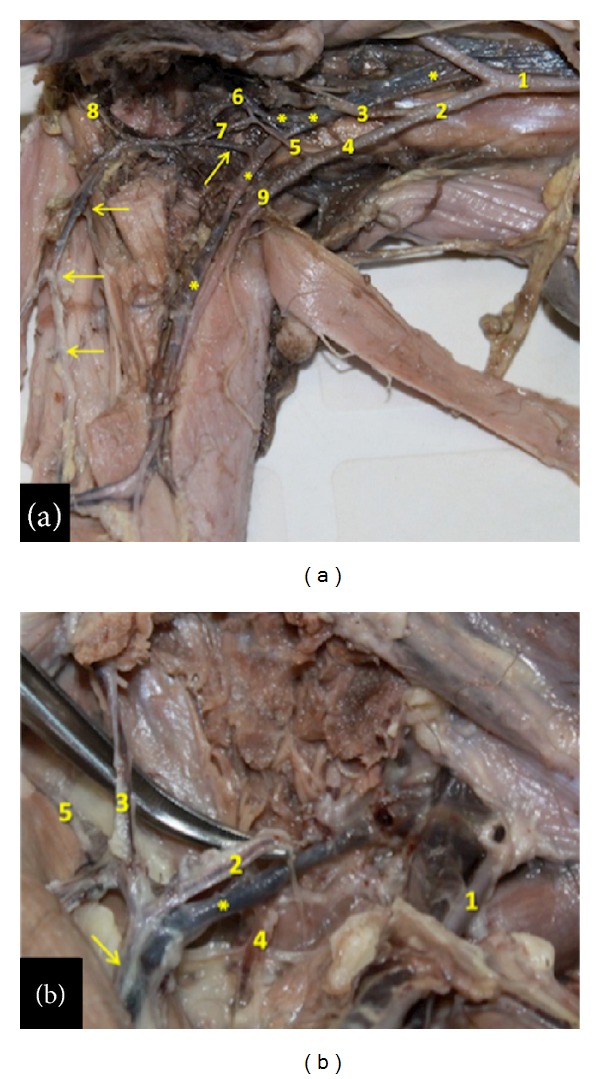
Ventral view of arteries in left hind limb of the bearded capuchin. Cranial to the right (a): View of the left pelvis and thigh. 1 indicates the abdominal aorta artery; 2 the left common iliac artery; 3 the internal iliac artery; 4 the external iliac artery; 5 the obturator artery; 6 the branch to the genital organ; 7 the medial circumflex femoral artery; 8 the external pudenda artery; 9 the femoral artery; the ∗ indicates the respective veins associated with arteries; the arrows indicate the saphenous vein (0.7X). (b) View of the left inguinal region. 1 indicates the femoral artery; 2 the medial circumflex femoral artery; 3 the muscular branch to the gracilis muscle; 4 the artery to the femur head; 5 the external pudenda artery. The arrow indicates the muscular branch to the semimembranosus muscle and the ∗ indicates part of the saphenous vein (2.4X).

In the bearded capuchins investigated, the external iliac artery runs inferior and laterally in the pelvis lying on the ilium-psoas muscle. It emits a medial-inferior branch, the obturator artery (Figure 1(a)), immediately before crossing the aponeurosis of the external oblique muscle of the abdomen. We did not find a true inguinal ligament in any of the specimens studied here. The obturator artery emits the medial circumflex femoral artery, which crosses the aponeurosis of the external oblique abdomen muscle medially to iliac vein (Figures 1(a) and 1(b)). In one specimen, however, the medial circumflex femoral artery originates directly from external iliac artery but only at one side.

The medial circumflex femoral artery crosses inferiorly to the aponeurosis of the external oblique abdomen muscle, runs obliquely from the anterior to the posterior aspect of the thigh, and communicates branches to the gracilis and adductor muscles, one single branch to the femur head and emits the external pudenda artery (Figure 1(b)). The medial circumflex femoral artery is usually a branch from the obturator artery.

**Figure 2:**
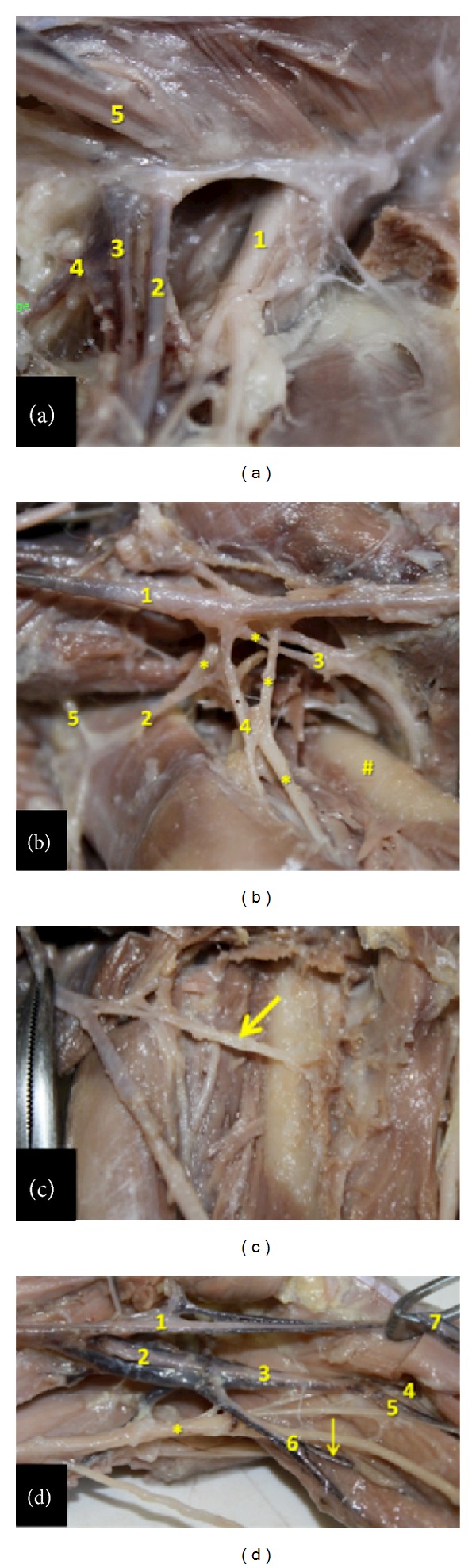
Detailed view of the inguinal, thigh, and popliteal regions in the bearded capuchin. (a) Left inguinal region. 1 indicates the femoral nerve; 2 the femoral artery; 3 the femoral vein; 4 the medial circumflex femoral artery; 5 the spermatic cord (3X). (b) Right thigh. The arteries are indicated by numbers, where 1 is the femoral artery; 2 the lateral circumflex femoral; 3 the profunda femoris artery; 4 the branch of the lateral circumflex femoral artery and 5 the inferior epigastric. The ∗ indicates veins associated with arteries; the # indicates the femur (2.4X). (c) Right thigh. The arrow indicates the profunda femoris artery (2X). (d) Left popliteal region. 1 indicates the femoral artery; 2 the popliteal artery; 3 the common tibial artery; 4 the anterior tibial artery; 5 the posterior tibial artery; 6 the fibular artery; 7 the saphenous artery. The arrow indicates a muscular branch from fibular artery to gastrocnemius and the ∗ indicates the sciatic nerve (1.8X).

In the bearded capuchin the femoral artery (Figures 1(a), 1(b), 2(a), and 2(b)) emits first a lateral trunk that gives rise to the lateral circumflex femoral artery and the superficial epigastric artery (Figure 2(b)). The superficial epigastric artery turns laterally and superiorly and emits, superior-laterally, the superficial circumflex iliac artery. The lateral circumflex femoral artery emits two muscular branches to the vastus lateral and vastus intermedius muscles, a descendent branch, and a branch that runs inferiorly and laterally towards the femur's head. We have identified the branches of the lateral circumflex femoral artery as the superficial epigastric artery (“ascendant branch”), and the lateral circumflex femoral artery (“descendent and transversal branches”).

In our specimens we observed that the first medial branch of the femoral artery is the profunda femoris artery that runs medial and posterior in the thigh (Figures 2(b) and 2(c)). It gives rise to muscular branches to the adductor muscles and quadriceps muscle in the medial side of the thigh, and one or two perforating branches that turn laterally. The profunda femoris artery terminates in muscular branches caudal in the lateral compartment of the thigh to the vastus lateralis muscle. The femoral artery continues in the thigh medially under the sartorius muscle through the adductor canal and inferiorly to the lower margin of the vastus medialis muscle, clearly giving rise to branches to the adductor muscles, the sartorius muscle and the vastus medialis muscle. In the distal quarter of the thigh the popliteal artery originates posteriorly and continues superficially as the saphenous artery (Figures 1(a) and 2(d)). In the bearded capuchins described here, the popliteal artery is a short vessel. It emits an ascendant trunk that gives off two inferior genicular arteries and later splits anteriorly into the common tibial artery and posteriorly into the fibular artery (Figure 2(d)).

**Figure 3:**
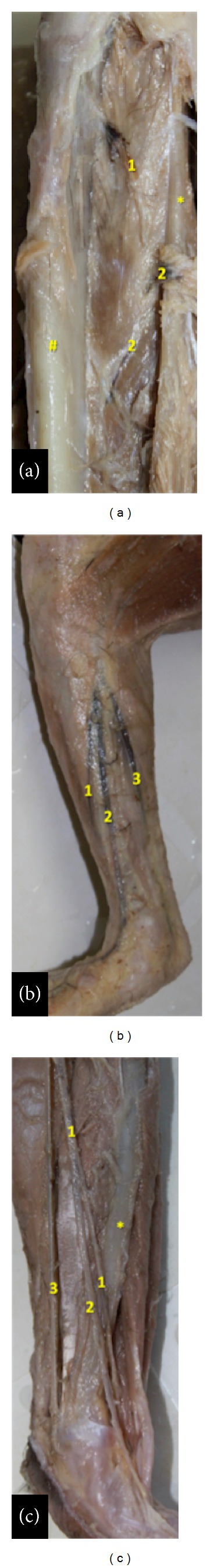
View of the lower leg of the bearded capuchin. Superior to the top. (a) Right leg. 1 indicates the anterior tibial artery and 2 the posterior tibial artery, both emerging in the anterior compartment of the leg. The # indicates the tibia and the ∗ the fibula (1.4X). (b) Right leg. The fascia of leg was kept and the arteries were not separated from the veins (0.8X). (c) View of the medial aspect of the left leg (1X). (b)-(c) The numbers indicate the branches from the saphenous artery. 1 indicates the anterior ramus; 2 the medium ramus; 3, the posterior ramus. The ∗ indicates the tibia.

The common tibial artery in the bearded capuchin (Figure 3(a)) divides into an anterior branch, the anterior tibial artery, and in the posterior and inferior branch, that is, the posterior tibial artery. The anterior tibial artery passes between the tibia and the fibula to turn proximately towards the anterior leg compartment and terminates into muscular branches supplying anterior superficial muscles of the leg (Figure 3(a)). The posterior tibial artery runs medially to the soleus muscle, lies at the posterior aspect of the interosseous membrane and penetrates in the middle third of the leg of this membrane to supply the deep anterior muscles of the leg in the anterior leg compartment (Figure 3(a)). The popliteal artery was considered here as the posterior tibial artery also due to position and function; the posterior and anterior tibial arteries do not reach the foot.

**Figure 4:**
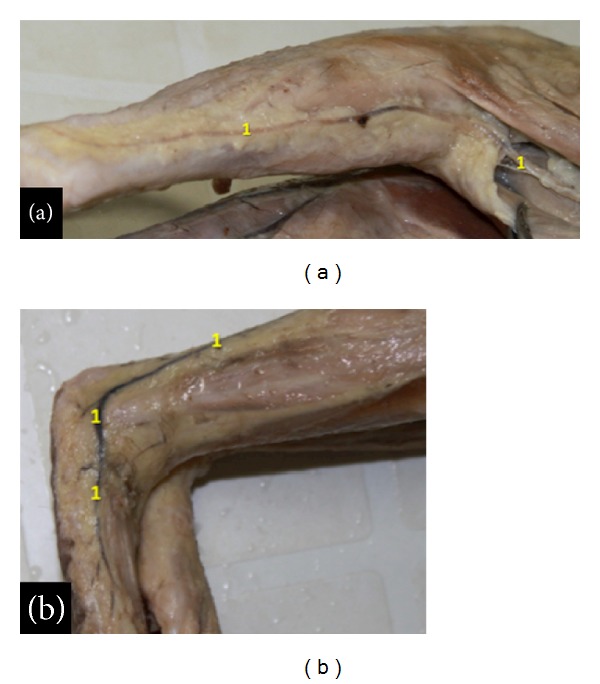
View of the posterior (a) and lower (b) leg of the bearded capuchin. The number 1 indicates the fibular artery that runs in the proximal leg together with the saphenous vein. (a) View of the posterior aspect of the left leg and part of the thigh (0.8X). (b) Lateral view of the lower leg and foot and part of the right leg (0.8X).

The fibular artery observed in the bearded capuchins (Figure 2(d)) is a branch from the popliteal artery. It gives rise to branches to the triceps sural muscles, continues superficially between the heads of the gastrocnemius muscle, and lies parallel to the short saphenous vein (Figure 4). Both, the fibular artery and the saphenous vein, run laterally to the gastrocnemius tendon along its lateral margin, passing posteriorly and inferiorly to the lateral malleolus into the foot's dorsal area. From there the arteries distribute laterally and superficially as a calcaneal branch.

The saphenous artery (Figure 3(b)) is superficial, emits the superior genicular arteries, and divides into the anterior and posterior branches in the medial proximal third of the leg. The anterior branch generates a medium branch in the proximal third of the leg (Figures 3(b) and 3(c)). Initially, all branches from saphenous artery run under the skin of the leg (Figure 3(b)). In the foot, they split into the dorsal and the plantar arteries. The anterior ramus of the saphenous artery (Figure 3(c)) runs, after giving rise to the medium ramus, inferiorly and penetrates into the distal third of the leg underneath the retinaculum of the anterior leg muscles. It passes then obliquely to the lateral aspect into the retinaculum behind the tendon of the anterior tibial muscle. From there it emerges laterally on the foot, where finally it emits the dorsalis pedis lateral artery (Figures 3(c) and 4(a)).

**Figure 5:**
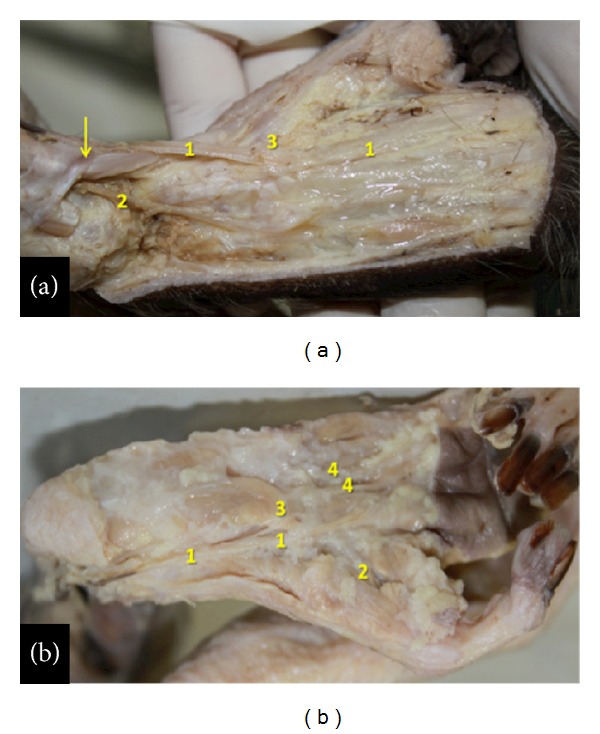
Right foot of the bearded capuchin. (a) Anterior and medium ramus of the saphenous artery on the dorsal foot. The number 1 indicates the dorsalis pedis medial artery; 2 the dorsalis pedis lateral artery; 3 the 1st dorsal metatarsal branch. The arrow indicates the retinaculum of the anterior leg muscles (2X). (b) Plantar view. The number 1 indicates the medial plantar artery; number 2 the hallux plantar artery; number 3 the lateral plantar artery; number 4 the 1st plantar metatarsal artery (2X).

The dorsalis pedis lateral artery distributes blood to soft tissues and skin and emits the 3rd and 4th dorsal metatarsal branches. The medium ramus from the anterior branch of the saphenous artery (Figure 3(c)) runs inferiorly, passes anteriorly to the medial malleolus, superficially to the retinaculum (Figure 4(a)), and emerges on the medial portion of the foot emitting the dorsalis pedis medial artery (Figure 5(a)) that gives off the 2nd dorsal metatarsal, a perforating branch and the first dorsal metatarsal artery. The posterior ramus of the saphenous artery (Figure 3(b)) runs inferior and medially along the medial margin of the medial head of the gastrocnemius muscle, continues medially to the tendon of the triceps sural muscle, and penetrates the foot inferiorly (Figure 5(a)) and medially becoming the medial plantar artery (Figure 5(b)). The medial plantar artery is associated with the plantar nerve in the sole (Figure 5(b)). It splits into a branch to the hallux (the first plantar metatarsal branch), a branch to the second interosseous space, and a communicating branch and gives off the lateral plantar artery approximately in the proximal third of sole. The lateral plantar artery (Figure 5(b)) supplies the third and fourth interosseous spaces.

### 3.2. Vein Description

The veins in the hind limb form a superficial and a deep group in the bearded capuchins. The deep veins are “venae comitantes,” that is, companion veins that follow like duplicates the arteries (Figures 1(a), 1(b), 2(d), and 3(b)). A highlighted superficial vein in the bearded capuchin is the short saphenous vein (Figure 4) that originates dorsal and laterally in the foot follows laterally and turns around the lateral malleolus. It ascends superficially between the heads of the gastrocnemius muscle together with the fibular artery, is superficial and posterior in the knee (popliteal fossa) and thigh, and continues accompanying the muscular branch of the medial circumflex femoral artery in the proximal third of thigh (Figures 1(a) and 1(b)).

The short saphenous vein continues to merge with the external iliac vein in the pelvis. The other superficial veins are a group formed by companion veins of the saphenous artery's branches that drain to the femoral vein, which represents the long saphenous. We found three superficial veins: two along each branch of the saphenous artery and a third one along the medium ramus of the anterior ramus of the saphenous artery.

The femoral vein receives the tributaries from veins accompanying the saphenous arteries. In the medial, aspect of the thigh of the bearded capuchin the femoral vein ascends passing into the femoral sheath, medial and deep to the femoral artery and lateral and anterior to the medial circumflex femoral artery and vein (Figure 2(a)). The external iliac and internal veins join to form the common iliac vein that drain to the inferior cava vena (Figure 2(a)).

## 4. Discussion

### 4.1. Comparison with Other Primates: Arteries (Table 1)

Interestingly, few variations were found among the specimens of bearded capuchin observed in the present study. The main variation, the origin of the medial circumflex artery in one of nine cases, only allowed a general description of the pattern of vessels in the bearded capuchin. We also found that the inguinal ligament in general extends between the spina iliaca anterior superior of the ilium and the tuberculum pubicum of the pubis and separates a space for muscles and vessels between abdomen and leg. In contrast to the description of Manners-Smith [19] in *Cebus* we could not find a true inguinal ligament in any of the specimens of bearded capuchin studied here.

The origin of the obturator artery in monkeys and apes is quite diverse: it was found to originate from the external iliac artery in *Cebus capucinus* and in *Pan* [19]; from the internal iliac artery in *Papio* and in ca. 50% of *Pan* [23, 24]; directly from the femoral artery or from the profunda femoris artery in 25% of *Papio* [25]; and from the inferior epigastric artery in *Gorilla*, *Pongo*, and in ca. 50% of *Pan* [23]. According to Gibbs [23], the obturator artery originates from the femoral artery as a variation in humans, while it originates from the profunda femoris, internal iliac [26], and inferior epigastric artery in 20 to 30% of all cases. The origin of obturator artery found here in bearded capuchin investigated here is similar to *Cebus* [19] and *Pan* [24]. However, we found in one specimen that the medial circumflex femoral artery originates on one side directly from external iliac artery.

Manners-Smith [19] described that the external iliac arteries gives off a common trunk to the inferior epigastric, obturator, and medial circumflex femoral artery in *C. capucinus*. Also, the latter emits branches to the adductor muscles and its deep portion passes between the pectineus and psoas major muscle. The medial circumflex femoral artery originates from the profunda femoris artery in *Homo* [23, 24, 26] and in 50% of the *Pan* [23], from the external iliac artery in 50% of *Pan* and *Hylobates*, and from the obturator artery in *Papio*. An origin directly from the femoral artery occurs in *Gorilla* and *Pongo* [23] but is also described as a variation in *Homo* [23, 26]. The origin of the medial circumflex femoral artery observed here in bearded capuchin is similar to *Papio* [24]. Nevertheless, the medial circumflex femoral artery is variable and may originate from external iliac, internal iliac, or obturator arteries in primates [24].

The external pudenda artery originates directly from the femoral artery in *Homo* [26] and *Pan* and unilaterally in *Gorilla* [23] *Papio* [24] and in 40% of the *Papio anubis* specimens [25]. Within the bearded capuchin we observed high variability in the origin of the external pudenda artery with every specimen having a different one, but it originates mainly from medial circumflex femoral artery. The origin of the external pudenda artery is different in all species reported in previous study.

In the *C. capucinus* specimens studied by Manners-Smith [19] the lateral circumflex femoral artery arises like an independent branch from the femoral artery and divides into ascendant, descendent, and transversal ramus. The inferior epigastric artery is described as originating from the medial circumflex femoral artery or from a trunk of the external iliac artery. Nonetheless, the superficial epigastric artery was not mentioned. The lateral circumflex femoral artery is a branch from the profunda femoris in all apes (except half of the *Gorilla*) and divides directly from femoral artery into ramus ascendant and descendent in *Gorilla* [23]. This is also described by Swindler and Wood [24], for *Pan*, *Papio* and *Homo* [26] although its origin in *Homo* may stem from the femoral and external iliac artery. In *Papio anubis* the lateral circumflex femoral artery originates from the femoral (35%) or the profunda femoris artery (65%) alone or in a common trunk with the medial circumflex femoral artery [25]. Gibbs [23] described that the lateral circumflex femoral artery might arise directly from femoral artery in *Homo*.

In *Gorilla* the superficial epigastric artery is a branch from the lateral circumflex femoral artery in *Pan* it is a branch from the external iliac artery, and it originates from the femoral artery in *Homo* and 50% of the *Pongo* [23, 26]. According to Swindler and Wood [24], the superficial epigastric artery originates directly from the external iliac artery and gives off the superficial iliac artery in *Papio*, *Pan* and *Homo*. In *Papio Anubis*, however, it originates always from femoral, either alone or in a trunk together with the external pudenda artery and/or the superficial circumflex iliac artery [25].

The description of the lateral circumflex femoral artery in the bearded capuchin in the present work is similar to that in Manners-Smith [19] and in some cases of *Homo* [24] and *Gorilla* [23]. Since Manners-Smith's account is relatively unclear regarding its branches, we have identified them as the superficial epigastric artery (“ascendant branch”), and the lateral circumflex femoral artery (“descendent and transversal branches”). The origin of the superficial epigastric artery is similar to *Homo* [23, 26] and 50% of the *Pongo* [23]. The superficial circumflex iliac artery was found to originate from the femoral artery in *Homo* and great apes [23, 26], from the inferior epigastric artery in *Homo*, *Pan*, and *Papio* [24, 26], but its origin was not described by Manners-Smith [19] in *Cebus*. Therefore, according to our data, the origin of the superficial circumflex iliac artery in bearded capuchins is similar to *Papio* and some of the apes and *Homo*.

According to Manners-Smith [19] the profunda femoris artery arises from the femoral artery, just below the lateral circumflex femoral, and gives off a branch to the adductor group and three perforating branches. The profunda femoris originates from the femoral artery and supplies the adductor muscles in all apes [23], *Papio* [24], and *Homo* [23, 24, 26]. It supplies the quadriceps in *Homo*, *Pan*, and *Pongo* and the hamstrings in *Homo* and *Pan*. The profunda femoris does not give rise to perforating branches in one third of *Pongo*, but emits two perforating branches in *Pan* and two-third of *Pongo* and three in *Homo* and *Gorilla* [23]. Nevertheless, it usually gives off four perforating branches in *Homo*, *Pan*, and *Papio*, according to Swindler and Wood [24]. Unfortunately, Dyl and Topol [25] did not describe the perforating branches of the profunda femoris in *Papio* anubis. The origin of the profunda femoris observed in our bearded capuchins is identical to the one in *Cebus* of Manners-Smith [19], *Homo*, and apes, but the number of perforating branches is more similar in *Pan* and in the majority of *Pongo*, according to Gibbs [23]; whereas in *Cebus* [19], the number of perforating branches is the same as in *Homo* and *Gorilla*.

Manners-Smith [19] describes in *Cebus* that the femoral artery runs in the adductor canal whence it emits branches to the adductor muscles and in the lower end of the canal divides into saphenous and popliteal arteries. Brown [18] (citing Meckel) stated that the femoral artery divides into two branches, a superficial muscular and a deep one. The latter supplies the anterior and posterior leg and probably represents the great saphenous artery, which divides into anterior small vessel that descends to dorsum foot and the posterior ones that descend to the sole. Gibbs [23] did not describe the trajectory of the femoral artery but mentioned that it has muscular branches t: the adductors in *Homo*, African apes, and *Hylobates*; to the vastus medialis muscle in *Homo* and African apes; and to the sartorius muscle in *Homo*. Swindler and Wood [24] did not mention the trajectory or division of the femoral artery in *Homo*, *Pan*, and *Papio*; but Dyl and Topol [25] found its division into popliteal and saphenous arteries in *Papio anubis*. The position and division of the femoral artery seem to be similar in all studied primates, as well as in the bearded capuchin observed here, except in *Homo* where the saphenous artery appears like a variation [19] or as a small branch from the descending genicular artery [23].

In *Cebus* [19], the popliteal artery gives off an anterior tibial and a rudimentary posterior tibial artery and continues as fibular artery. It gives rise to the superior and medial genicular arteries. The popliteal artery lies deep in the popliteal fossa only in *Homo*. In all apes and *Homo*, it originates from the femoral artery, except in *Pan* where it divides into posterior tibial artery and a common branch for the anterior tibial and fibular arteries [23, 24]. In *Homo*, the popliteal artery is a continuation of the femoral artery. It emits the superior, middle, and inferior genicular arteries [23] and divides at the level of the proximal end of the crural interosseous space into tibial anterior and posterior arteries, in 90% of the cases [26]. Nevertheless, Swindler and Wood [24] described that the femoral artery gives off the descending genicular artery before it passes to popliteal fossa to become popliteal artery and that the saphenous artery is a thin and insignificant end branch of the descending genicular artery placed in the medial side of knee and leg. In all apes and *Homo*, the popliteal artery gives rise to the superior, medial, (except in *Hylobates*) and inferior genicular arteries (except in *Gorilla* and *Pongo*) [23].

With respect to the branches of the popliteal artery, the pattern observed in the bearded capuchins is different from other primates described so far, but it is identical to the description by Manners-Smith for *Cebus* [19]. Despite that, the nomenclature employed in the present work is different; that is, the “rudimentary posterior tibial artery” of Manners-Smith [19] is described here as posterior tibial artery and, since the “superior genicular artery” branches off inferiorly to the other genicular branch of saphenous artery, it was identified here, in bearded capuchin, as the inferior genicular artery. Furthermore, the main difference of the popliteal artery branches in bearded capuchins to other primates lies in its division into common tibial and fibular arteries, whereas the majority of the apes (except to *Pan*) present a division into anterior and posterior tibial arteries. Gibbs [23] described a division of the popliteal artery into anterior tibial and fibular arteries as a variation in *Homo* that is somewhat similar to the one observed here in the bearded capuchin.

Manners-Smith [19] did not describe in detail the distribution of anterior tibial artery and his “rudimentary posterior tibial artery” for *Cebus*. In *Homo*, *Pan*, and *Pongo*, the anterior tibial artery passes between tibia and fibula; it emits the fibular artery in *Pan* and *Pongo* and reaches the foot in *Homo* and *Pan* but not in *Pongo* and *Gorilla*, where it may be replaced distally by the saphenous artery. This replacement seems to appear in great apes [23], similarly to what has been observed here in bearded capuchins. The fibular artery arises from posterior tibial artery in *Homo* [23, 24, 26]. The posterior tibial artery is the terminal branch of popliteal artery in all apes, except in *Pan*; it branches off into medial and lateral plantar arteries in *Pongo* and *Gorilla* but not in *Hylobates*, *Pan*, and *Homo* [23, 24, 26].

In *Papio*, Manners-Smith [19] described that the popliteal artery divides into anterior tibial and fibular arteries, but Swindler and Wood [24] mentioned a large branch from popliteal artery that they called the posterior tibial artery due to its position and function. Indeed, in the bearded capuchin investigated here, a branch from popliteal artery was considered as the posterior tibial artery also due to position and function, in keeping with Swindler and Wood regarding the true posterior tibial artery. In the bearded capuchin, posterior and anterior tibial arteries do not reach the foot, similarly to the description in *Papio* where these vessels end near to ankle.

The trajectory of the fibular artery was not descripted by Manners-Smith [19] for *Cebus*. The fibular artery is a branch from the posterior tibial artery in *Homo*, *Gorilla*, and *Hylobates*, from the anterior tibial artery in *Pongo* and from the popliteal artery in *Pan* [23, 26]. Interestingly, according to Swindler and Wood [24], the fibular artery is a large branch from the posterior tibial artery in *Pan*, which is in disagreement with Gibbs [23], but this could also be just a variation from different studied specimens. The fibular artery gives rise to perforating and lateral calcaneal rami in all apes and *Homo* [23]. The origin of the fibular artery in the bearded capuchin and *Cebus* [19] is similar to *Pan* [23], but the distribution is similar to all apes and *Papio*.

Manners-Smith [19] mentioned the saphenous artery in *Cebus*, which gives rise to the suprema genicular artery and splits into the anterior (dorsal) and posterior (plantar) branch. The dorsal branch was called posterior tibial artery (see above). The plantar artery divides into superficial and deep branches. This description is similar to our observation in the bearded capuchin regarding the region of the distribution, although different names and position were ascribed. With respect to the position, there seems to be a discrepancy in the *Cebus* descriptions of Manners-Smith [19]: the dorsal division was first identified as “tibialis posterior” (page 120), and later the plantar division was also identified as “tibialis posterior” (page 121). The latter case seems to be correct as the dorsal division is associated with the posterior ramus of the saphenous.

The superficial part of the anterior division of saphenous artery subdivides into inner and outer branches according Manners-Smith [19]. The inner branch is continuous as first dorsal metatarsal artery and communicates with arteries of the sole. The outer branch joins with the perforating branch of the fibular artery to form an arch. The deep part also forms an arch that emits the dorsal metatarsal arteries in *Cebus*. The posterior (plantar) division of the saphenous artery enters into the plantar region and divides into the lateral plantar and medial arteries. The saphenous artery runs medially to the knee in great apes and *Homo* and anastomoses with the medial inferior genicular artery, but in great apes it runs together with the saphenous nerve to the foot as dorsalis pedis artery and penetrates the first interosseus space to form the plantar arch [23].

Swindler and Wood [24] described also two branches from saphenous artery, the anterior and posterior, both supplying the distal leg and foot in *Papio*. In *Pan* there is a large saphenous artery to supply the dorsal foot and the sole is supplied by the posterior tibial artery. According to the authors cited here, the medium ramus from the anterior branch of the saphenous artery seems to be the dorsalis pedis artery. In this work, however, to bearded capuchin, the name “dorsalis pedis” was only used when this artery was located in the foot. In the bearded capuchin, we did not observe the perforating branch from the fibular artery and its communication with the anterior branch of the saphenous. Except for that, the rest of the description is similar to *Cebus* from Manners-Smith [19].

In the bearded capuchin the dorsalis pedis lateralis is a branch from the anterior ramus of the saphenous artery and the dorsalis pedis medialis is a branch from the ramus medialis of the anterior ramus of the saphenous artery (present work). In *Cebus* according to Manners-Smith [19], the dorsalis pedis lateralis is a branch from the deep division of the saphenous artery. The dorsalis pedis medialis, on the other hand, is a division from the inner branch of the anterior division of the saphenous artery and therefore was identified as first dorsal metatarsal artery. The dorsalis pedis artery is the final branch of the anterior tibial artery in *Homo* [26], posterior tibial artery in *Pan*, the continuation of the saphenous in great apes, and completes the plantar arch in *Homo*, *Pan*, and *Pongo* [23]. Therefore, our description of the dorsalis pedis artery in the bearded capuchin is more similar to *Cebus* and *Papio*.

In *Homo* and *Pan* the medial plantar arteries supply the first to third interosseous spaces and in *Pongo* the second and third ones. The lateral plantar artery is absent in *Hylobates*. It crosses the sole obliquely and emits a communicating branch to the dorsalis pedis artery to complete the plantar arch in great apes and *Homo* [23]. The origin of the plantar arteries in the bearded capuchins is similar to *Cebus* and *Papio*.

**Figure 6:**
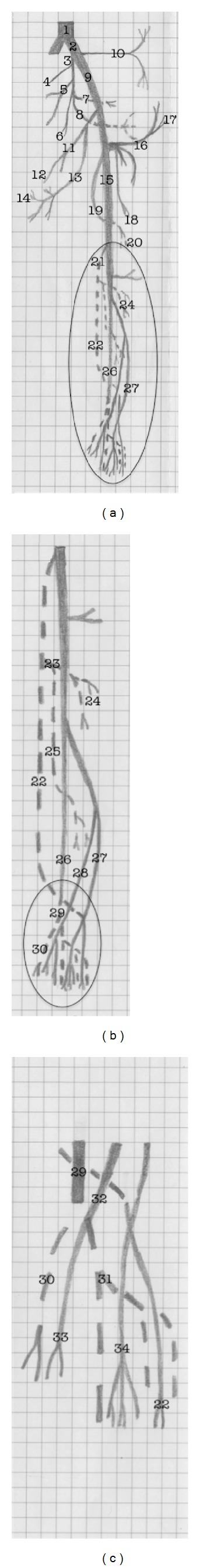
Schematic representation of the arterial pattern of the hind limb of the bearded capuchin. (a) General scheme. (b) Details of the circle in (a), that is, leg's scheme. (c) Details of the circle in (b), that is, foot scheme. The dashed line indicates a position behind the plane and continuous line represents the plane level. The numbers indicate the names of arteries. 1 the aorta; 2 the common iliac; 3 the internal iliac; from 4 to 8 indicate the pelvic arteries not named in this work; 9 the external iliac; 10 the iliolumbar artery; 11 and 12 the obturator artery and its muscular branch; 13 the medial circumflex artery; 14 the external pudenda artery; 15 the femoral artery; 16 the inferior epigastric artery; 17 the superficial circumflex iliac artery; 18 the lateral circumflex femoral artery; 19 the profunda femoris artery; 20 the perforating branch; 21 the popliteal artery; 22 the fibular artery; 23 the common tibial artery; 24 the anterior tibial artery; 25 the posterior tibial artery; 26 the posterior saphenous branch; 27 the anterior saphenous branch; 28 the medium saphenous branch; 29 the medial plantar artery; 30 the first plantar metatarsal branch; 31 the lateral plantar artery; 32 the medial dorsal artery; 33 the first dorsal metatarsal artery; 34 the lateral dorsal artery.

In general, the pattern of arteries of the bearded capuchin hind limbs (Figure 6) is more similar to *Cebus* described by Manners-Smith [19] (Table 1). However, we observed specific differences in relation to origins, distribution, and names (see above discussion). The most likely explanation is that Manners-Smith [19] analyzed capuchin from Central America (*Cebus* genus) whereas we investigated the bearded capuchin from South America, which has been recently segregated as independent genus (*Sapajus*). On the other hand, as discussed by Swindler and Wood [24], problems in the interpretation were generated by the differences in nomenclature as for example, the posterior tibial artery to the posterior branch of the saphenous. Finally, other small differences could be explained by natural variation among specimens.

**Figure 7:**
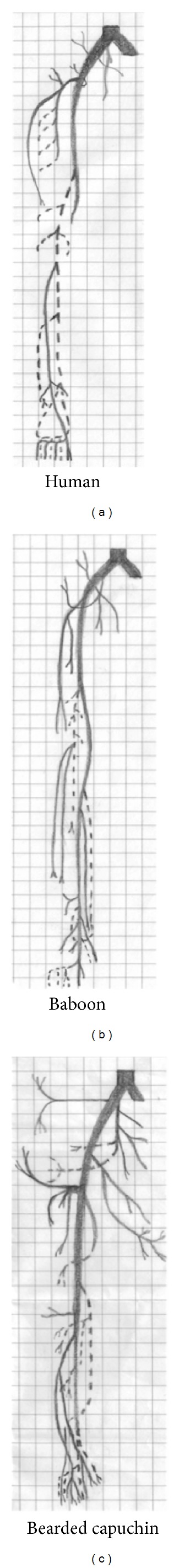
Schematic representation of the arterial pattern of the hind limb of the modern human and baboons, based on Swindler and Wood [24], and the bearded capuchin.

The arterial pattern observed for the hind limbs vessels in the bearded capuchin is somewhat similar to model observed in *Papio*. The similarities are probably due to a comparable pelvis and the presence of a tail. The characters become more different in great apes and *Homo* (Figure 7). Despite that, the anatomical descriptions in nonhuman primates are scarce and most of them are not thorough, rendering complete comparisons impossible. Future studies could focus on comparative analysis among bearded capuchin and other New World monkeys to supply data for taxonomy, phylogeny and evolution of apes and monkeys.

### 4.2. Comparison with Other Primates: Veins

According to Gibbs [23] in *Gorilla* the short saphenous vein splits into two branches, both of which merge with the popliteal vein in the popliteal space. It is a lateral vein in the human leg and one of two lateral veins in *Pongo*'s leg [23]. The short saphenous vein begins on the lateral foot in *Papio*, runs in the lateral side of the thigh, and drains into the popliteal vein [24]. Different from the observations in baboons, apes, and *Homo*, the short saphenous vein in the bearded capuchins continues to merge with the external iliac vein in the pelvis. The other superficial veins in the bearded capuchins are the group formed by companion veins of the saphenous artery's branches that drain to the femoral vein, which represents the long saphenous. Gibbs [23] described one medial superficial vein in *Homo*, *Pongo*, and *Pan* and two in *Gorilla* as the long saphenous. Bearded capuchins on the other hand present three superficial veins: two along each branch of the saphenous artery and a third one along the medium ramus of the anterior ramus of the saphenous artery.

### 4.3. Comments on Bipedalism

The accurate studies considering the diameter of the vessels and number of branches of arteries to muscles and to compare them among the various primates was no yet made and could present many difficulties to be performed; therefore, the anatomical data about arterial distribution can give the more objective information to discuss deeply all subjects under the bipedalism evolution. In fact, it has been recently argued that the adoption of incremental terrestriality by arboreal primate species may have been crucial to the development of tool use in primates [27]. Terrestrialism does not equate to bipedalism in terms of the conditions for complex manipulation, although as noted before, bipedalism does substantially spare the upper limbs from the locomotory function. Arguably, terrestriality without bipedalism could still be advantageous for the arboreal species since less effort is necessary to maintain balance on the ground and consequently one or both forelimbs could be freed for the handling of objects.

Capuchins are quadrupeds in strict sense [28] but switch easily from quadrupedal to bipedal postures and thus may be an important anthropological model for the evolution of human bipedalism [2]. Interestingly, the intermittent bipedalism observed in the bearded capuchin is a strenuous or at least difficult activity [17], specially when coupled with other activities, such as nut-cracking [29]. More morphological studies are required to provide important insights into this behavior as well as to associate behavior and morphology of the primate musculoskeletal system, to improve the understanding of the bipedalism evolution in the order primates. Indeed, most of the morphological studies in order to understand bipedalism in primates are focused on the musculoskeletal apparatus. However, the vascular supplement to muscles could play an important role in the aspect as they supply the muscles needed for the bipedal gait.

Indeed, the aspect to be considered is the relation between volume of muscle, that is, the tissue to be supplied, and the surface covered by vessels that supply these muscles. The surface-to-volume ratio plays an important role in the metabolism in general and to muscles physiology and this might depend on the volume of body or an organ. In fact, the capuchins must have higher relative superficial area than other larger primates, and it could explain the high muscles capacity to support the weight of animal when in bipedal gait; however, this knowledge is not enough to generate a conclusion on the special behavior about the *Sapajus* bipedalism rather than others primates with same proportions. Interestingly, occasional bipedal gait has been observed in several primate species; few of them adopt this posture while handling tools. This is the case even for closely related species, for example, *Cebus albifrons*, *C. olivaceus*, *C. capucinus*, and *Sapajus libidinosus* have all been shown to engage in bipedal locomotion, but this behavior has only been observed in association with tool use in the *Sapajus*.

Probably, as small-bodied primates, all capuchin species would benefit from the higher surface-to-volume ratio in hind limb vessels to stay in a bipedal gait. Since only a few such species present prolonged bipedal posture during tool use, higher surface-to-volume may be a necessary but not sufficient condition for occasional bipedalism in capuchins. Moreover, the results presented here indicate that the bearded capuchin has no specific anatomical circulatory apparatus for this behavior, because it was not possible to analyze whether the *Sapajus libidinosus* has more arterial ramification than others studied primates or to verify the diameter of the arteries comparatively. Indeed, there are no data available in literature that would specifically relate to this question.

## 5. Conclusions

Compared to all primates included in the present study, the arterial pattern observed in bearded capuchins is closest to the description of *Cebus capucinus* made by Manners-Smith [19]. There are however a few differences regarding the origin, trajectory, and branching of the tibial artery, which may support the recent *Cebus*/*Sapajus *separation [5]. Bearded capuchins also show a high degree of similarity to baboons, which is probably due to the presence of tails in both genera. As expected from the phylogeny, the degree of similarity to apes and humans is lower. Nevertheless, the pattern of superficial veins observed in bearded capuchins is not similar to any of the primates in comparison, namely, the short saphenous does not drain to popliteal but to extern iliac vein. The pattern of the deep veins, on the other hand, shows very few discrepancies among all studied primates. As a whole, the hind limb vessels observed in the bearded capuchin do not seem to have any fundamental influence on the intermittent bipedalism displayed by these primates in relation to other primates with similar proportions. Future studies should focus on comparative anatomical analysis among capuchin genera and other New World monkeys, apes, and monkeys to responds fundamental questions as the interesting behavior associated to *Sapajus*.

## Figures and Tables

**Table 1:** Comparative anatomy of the hind limb arteries of bearded capuchin (this work), *Cebus capucinus*, baboons, apes, and *Homo*. O originates from E: emits.

	Bearded capuchins		*Cebus *		Baboons		Apes		*Homo *	
	O	E	O	E	O	E	O	E	O	E
Obturator	External iliac	Medial circumflex femoral muscular branches	External iliac	—	Internal iliac	—	External iliac (*Pan* [24]), internal iliac (50% of *Pan* [23]), inferior epigastric (*Gorilla*, *Pongo*, and 50% of *Pan* [23])	—	Originates from femoral as variation [23], profunda femoris, internal iliac [26], and inferior epigastric from 20 to 30%	Iliac branches to fossa iliac and anastomoses with iliolumbar artery [26]

Medial circumflex femoral	Obturator artery or external iliac (one case observed)	Muscular branches, femur head branch, external pudenda	External iliac	Muscular branches	Obturator	—	External iliac (*Hylobates* [23] and *Pan* [24]), profunda femoris (50% of *Pan* [23]), femoral (*Gorilla* and *Pongo* [23])	—	Profunda femoris [23, 24, 26] femoral as variation [23, 26]	Muscular branches [26]

External pudenda	Medial circumflex femoral	—	—	—	Femoral	—	Femoral (*Pan*, unilaterally in *Gorilla*; [23])	—	Femoral [23, 26]	Anastomose with branches of internal pudenda artery [26]

Femoral	External iliac	Lateral circumflex femoral, inferior epigastric, profunda femoris, popliteal, saphenous, muscular branches	External iliac	Lateral circumflex femoral, profunda femoris, saphenous, popliteal	External iliac	Saphenous, popliteal, profunda femoris, suprema genicular	External iliac [23]	Medial circumflex femoral (*Gorilla*, *Pongo*, as a variation in *Homo* [23]), descendent genicular (*Pan*, * Pongo*, *Homo* [23]), saphenous (all apes [23]), deep circumflex iliac (African apes; [23]), muscular branches [23], superficial circumflex iliac (great apes and *Homo* [23]), inferior epigastric (*Pan*, SW and all apes and *Homo*; [23]), external pudenda (*Homo*, *Pan*, *Gorilla* [23]), profunda femoris (all apes and *Homo*, except *Gorilla,* G), popliteal (all apes and *Homo,* G)	External iliac [23, 26]	Medial circumflex femoral (as a variation), descendent genicular, muscular branches, superficial circumflex iliac, inferior epigastric, external pudenda, profunda femoris, popliteal [23, 26]

Superficial epigastric	Femoral	Superficial circumflex iliac	—	—	External iliac	—	Lateral circumflex femoral (*Gorilla* [23]), femoral (50% *Pongo* [23]), external iliac (*Pan* [23])	—	Femoral [23, 26]	—
Lateral circumflex femoral	Femoral	Muscular branches, descendent branch, femur head branch	Femoral	Ascendant, descendent, and transversal	Profunda femoris	Ramus descendens	Profunda femoris (all apes, except 50% *Gorilla* [23]), femoral (*Gorilla* to ascendant and descendent ramus [23])	Three branches in *Pan* and Asian apes, five branches in* Pongo* [23]	Femoral, external iliac, [24] profunda femoris [23, 24, 26]	Three branches [23, 26]

Superficial circumflex iliac	Inferior epigastric	Muscular branches	—	—	Inferior epigastric	—	Inferior epigastric (*Pan* [24]), femoral [23]	—	Inferior epigastric [24], femoral [23]	—

Profunda femoris	Femoral	Muscular branches, perforans (1 or 2)	Femoral	Three perforans branches	Femoral	Perforans	Femoral [23]	Muscular branches, two perforans branches in 66.6% of *Pongo*, *Pan,* and *Hylobates*, three perforans branches in *Gorilla* [23]	Femoral [23, 26]	Three perforans branches [23, 26], lateral and medial circumflex femoral arteries and muscular branches [26]

Popliteal	Femoral	Inferior genicular, common tibial, fibular	Femoral	Anterior tibial, fibular, superior genicular, rudimentar posterior tibial, media genicular	Femoral	Anterior and posterior tibial arteries	Femoral [23]	Anterior and posterior tibial arteries in all apes, except *Pan* that divides into a posterior tibial and a common branch for the anterior tibial and fibular artery, superior genicular branches in all apes and inferior genicular branches in *Pan* and *Hylobates* [23]	Femoral [23, 26]	Anterior and posterior tibial arteries, superior genicular and inferior genicular branches, sural arteries [23, 26], middle genicular arteries, muscular branches, cutaneous branches [26]

Superior genicular	Saphenous	—	Popliteal	—	Popliteal	—	Popliteal [23]	—	Popliteal [23, 26]	—

Inferior genicular	Popliteal	—	—	—	Popliteal	—	Popliteal in *Pan* and *Hylobates* [23]	—	Popliteal [23, 26]	—

Anterior tibial	Common tibial	Muscular branches	Popliteal	—	Popliteal	—	Popliteal [23]	Muscular branches in all apes, except *Pan * are terminating as dorsal artery of the foot [23]	Popliteal [23, 24, 26]	Dorsal artery of the foot [23, 26], posterior tibial recurrent, anterior tibial recurrent, muscular branches, perforating branches, anterior medial malleolar, anterior lateral malleolar [26]

Posterior tibial	Common tibial	Muscular branches	Saphenous (from popliteal to rudimentaris posterior tibial)	Medial and lateral plantar	Popliteal	—	Popliteal [23, 24]	Calcaneal in great apes [23], medial, and lateral plantar [23, 24]	Popliteal [23, 24, 26]	Calcaneal, medial, and lateral plantar [23, 26], circumflex fibular, nutrient artery of the tibia, muscular branches, perforating branches, communicating branch, medial malleolar [26]

Fibular	Popliteal	Muscular branches, calcaneal	Popliteal	—	Popliteal	—	Posterior tibial in *Gorilla* and *Hylobates*, anterior tibial in *Pongo*, popliteal in *Pan* [23]	Lateral calcaneal, perforating branch in all apes [23]	Posterior tibial [23, 24, 26]	Lateral calcaneal, perforating branch in all apes [23, 26], muscular branches, nutrient to fibula, perforating branches, communicating branch [26]

Saphenous	Femoral	Superior genicular, anterior and posterior branches	Femoral	Suprema genicular, anterior, and posterior branches	Femoral	Anterior and posterior branches	Femoral	Dorsalis pedis in great apes [23]	Descending genicular [23]	—

Anterior branch of saphenous	Saphenous	Medium branch, dorsalis pedis lateral, 3rd and 4th dorsal metatarsal	Saphenous	—	Saphenous	—	—	—	—	—

Posterior branch of saphenous	Saphenous	Medial plantar	Saphenous	Medial and lateral plantar	Saphenous	Medial and lateral plantar	—	—	—	—

Medium branch of the anterior branch of saphenous (dorsalis pedis)	Anterior branch of saphenous	Dorsalis pedis medial, 1st dorsal metatarsal, 2nd dorsal metatarsal	Anterior branch of saphenous	1st dorsal metatarsal	Anterior branch of saphenous	Dorsal	Posterior tibial in *Pan*, saphenous in great apes [23]	—	Anterior tibial [23, 26]	Tarsal, arcuate, first dorsal metatarsal, cutaneous [26]

Medial plantar	Posterior branch of saphenous	To hallux, to second interosseous space, a communicating branch, lateral plantar	Posterior branch of saphenous	—	Posterior branch of saphenous	—	Posterior tibial in all apes [23]	From first to third interosseous spaces in *Pan*, from second to third ones in *Pongo *[23]	Posterior tibial [23]	Muscular branches, communicating branch [26]

Lateral plantar	Medial plantar		Posterior branch of saphenous	—	Posterior branch of saphenous	—	Posterior tibial in great apes [23]	Communicating branch [23]	Posterior tibial [23]	Muscular branch [26]
